# Time-varying associations between loneliness and physical activity: Evidence from repeated daily life assessments in an adult lifespan sample

**DOI:** 10.3389/fpsyg.2022.1021863

**Published:** 2023-01-26

**Authors:** Tiana Broen, Yoonseok Choi, Elizabeth Zambrano Garza, Theresa Pauly, Denis Gerstorf, Christiane A. Hoppmann

**Affiliations:** ^1^Department of Psychology, The University of British Columbia, Vancouver, BC, Canada; ^2^Department of Psychology, University of Zurich, Zurich, Switzerland; ^3^Department of Psychology, Humboldt University of Berlin, Berlin, Germany

**Keywords:** physical activity, loneliness, repeated daily assessments, lifespan, moderate-to-vigorous physical activity, number of steps, COVID-19, time sampling

## Abstract

Physical activity is a behavior that promotes physical and mental health; yet physical activity has decreased during the COVID-19 pandemic. To promote health during times of challenge, it is important to identify potential barriers to this key health behavior, such as loneliness. This brief report extends previous research on physical activity and loneliness that mainly focused on between-person differences to examine their time-varying associations at the within-person level using repeated daily life assessments. From April 2020 to August 2020, data were collected from a sample of 139 community-dwelling Canadian adults (*M*_age_ = 40.65 years, *SD* = 18.37; range = 18–83 years). Each evening for 10 consecutive days, participants reported their loneliness, number of steps, and minutes of moderate-to-vigorous physical activity. Results revealed that, in line with our hypotheses, on days when participants reported more loneliness they also engaged in less moderate-to-vigorous physical activity than on less lonely days (*estimate* = −0.24, *p* = 0.007); there was a significant negative association between loneliness and daily number of steps (*estimate* = −18.42, *p* = 0.041). In contrast, at the between-person level, overall loneliness was not associated with overall physical activity engagement after accounting for within-person differences and control variables (age, sex, day in study). From an intervention perspective, our findings suggest that it is promising to tackle loneliness on a day-to-day basis to increase physical activity one day at a time. This may be especially relevant during times mandating social-distancing, but also at other times when individuals experience greater feelings of loneliness.

## Introduction

Times of challenge that threaten physical and mental well-being, such as the COVID-19 pandemic (Creese et al., 2020; Wettstein et al., 2021; Zheng et al., 2021), highlight the importance of safe everyday behaviors that individuals may engage in to maintain their health (Holmes et al., 2020; Adams et al., 2021). Physical activity is one health-promoting behavior that has been encouraged by health officials during the pandemic as it has the potential to be implemented into daily routines according to social distancing measures (CDC, 2021). Even pre-pandemic, only 20% of adults worldwide engaged in the recommended 150–300 min of moderate or 75–150 min of vigorous physical activity per week (Geneva: World Health Organization, 2020) and emerging research indicates that physical activity behaviors have decreased significantly during the pandemic (Adams et al., 2021; Maltagliati et al., 2021; Shiba et al., 2022). The current brief report aims to better understand salient barriers to physical activity in day-to-day life to support well-being during times when “normal” life is interrupted by extenuating circumstances.

Loneliness is one barrier to physical activity that has become particularly salient during the restrictions of the COVID-19 pandemic (Ernst et al., 2022; Holt-Lunstad and Perissinotto, 2022). Psychological research defines loneliness as the subjective perception of lacking desired social contact and maintains that it is conceptually distinct from feelings of poor social support, perceived stress, depression, or hostility (Cacioppo et al., 2015). Elevated loneliness is associated with negative health outcomes, such as depression and anxiety symptoms (Pels and Kleinert, 2016), and it is as a risk factor for mortality (Holt-Lunstad et al., 2015). Hawkley and Cacioppo (2010) propose a “loneliness loop” model in which social isolation triggers expectations for negative social interactions, which then feed into a self-fulfilling prophecy whereby lonely individuals continue to isolate themselves and exhibit poorer emotional self-regulation, experience fewer positive emotions, and experience lower social control. Together, these consequences of loneliness contribute to a lower motivation for physical activity engagement (Hawkley et al., 2009).

Unsurprisingly, several recent studies have indicated that self-reported loneliness has increased during the COVID-19 pandemic (Elran-Barak and Mozeikov, 2020; Lee et al., 2021; O’Sullivan et al., 2021; Son et al., 2021). In line with Hawkley and Cacioppo’s (2010) “loneliness loop” model, recent research found a relationship between home confinement during the pandemic and lower prevalence of physical activity (Shiba et al., 2022). Research also indicates that loneliness has a trait and a state component such that loneliness varies both within-and between-persons (Hawkley et al., 2009; van Roekel et al., 2018). This, with the understanding that physical activity engagement is supported by social engagement and social support (Booth et al., 2000; Leinberger-Jabari et al., 2021), begs the question as to whether there are associations between day-to-day fluctuations in loneliness and daily physical activity behaviors, especially during times of challenge.

Much of the research examining physical activity as a health behavior looks at between-person differences (Creese et al., 2020; Maltagliati et al., 2021); however, repeated daily life assessments from community-dwelling samples also show that there is significant variation in physical activity behaviors at the within-person level (Pauly et al., 2020). Given that we cannot draw within-person conclusions based on between-person differences, analysis of within-person relationships allow us to examine more nuanced and immediate effects of day-to-day barriers to physical activity on physical activity engagement (Kanning et al., 2013). As individuals differ in day-to-day affective states and situational circumstances related to physical activity (Kanning et al., 2013), there is a need to better understand what differentiates a good day from a bad day.

To maximize ecological validity and identify targets for real-world intervention, this brief report uses repeated daily life assessments (Bolger et al., 2003). This measurement-intensive design allows us to capture variation in one’s thoughts, feelings, and behaviors across several days. Embracing the meaningful insights gained from getting a snapshot into one’s daily life, this method is employed to better understand associations between loneliness and physical activity during times of social distancing.

Embracing that physical activity is shaped by social contexts and that there is significant variation in physical activity on a day-to-day basis (Pauly et al., 2020), this study aimed to extend research on between-person differences in physical activity by examining loneliness on active vs. less active days. This study was launched in April 2020, shortly after the onset of the COVID-19 pandemic, and participants completed daily questionnaires for 10 consecutive days to get a snapshot of their thoughts, feelings, and behaviors during these unique times. We hypothesized that individuals who report more loneliness on a particular day will take fewer steps or engage in less moderate-to-vigorous physical activity that same day as compared to less lonely days. All analyses controlled for variables known to be associated with physical activity, including age, sex, and weekday versus weekend effects (Bellettiere et al., 2015; Burchartz et al., 2022). This project and associated hypotheses were pre-registered on Open Science Framework and can be accessed at the following link: https://osf.io/dvqrt/?view_only=bd39c2a276c84b27aefe77bb7bb64df3I.

## Materials and methods

### Participants

A total of 139 Canadian residents (*M*_age_ = 40.65 years, *SD* = 18.37, range = 18–83) were included in the final analyses of this project (for further details, see Pauly et al., 2021; Choi et al., 2022; Zambrano et al., 2022). The sample self-identified as 80% female, 73% white, 60% having a university degree, and being generally healthy (*M*_health_ = 3.32, *SD* = 0.94; on a 1–5 scale). Most participants lived with at least one other person (*M*_household size_ = 2.71, *SD* = 1.57), 35% reported raising children, 47% self-reported as not single, and 40% reported being employed at the time of the study. Out of the original 169 participants who completed the daily diary portion of this study, five were removed because they did not provide sex or age data, 24 were removed for completing only one evening questionnaire, and two were removed for never reporting any physical activity data. Participants were recruited through online advertisement, newspaper outlets, and via past participant pools. Participants were eligible for the study if they were 18 years or older, living in Canada, and had access to a computer or mobile device with internet connection to complete the questionnaires. The study was approved by the UBC ethics board [certificate number: H17-01249], and all participants provided informed consent.

### Procedure

From April 2020 to August 2020, data were collected online. Eligible participants received a link via email to complete the baseline questionnaire, which involved questions on sociodemographic characteristics, social and personality constructs, physical activity behaviors, and questions specific to the COVID-19 pandemic context. Following the initial questionnaire, participants were invited to complete brief online morning and evening questionnaires for 10 consecutive days, involving questions on affect, context, and activities that day. Participants were asked to self-report their daily number of steps and minutes of moderate-to-vigorous physical activity each evening by either estimating or, if available, using data from their personal fitness watch or smartphone. The average participant completed 6.70 evening questionnaires (*SD* = 2.81), and 73% of participants completed 5 or more evening questionnaires.

All participants were entered to win a $50.00 Amazon gift card for completing the initial questionnaire (1:10 chance of winning), and then entered again for completing 80% of the daily questionnaires (1:5 chance of winning). All procedures were conducted virtually, via email and the Qualtrics survey platform.

### Measures

#### Physical activity

Physical activity was operationalized using number of steps and minutes of moderate-to-vigorous physical activity. During the baseline questionnaire, physical activity was also assessed by self-report at the person-level. Participants were asked to reflect on: “How much time do you typically engage in high to moderate physical activity (the type that makes your heart beat more) on an average day?” (*M*_pre-pandemic_ = 54.76 min/day, *Median*_pre-pandemic_ = 40.00, *SD* = 52.37), referring to the amount of activity they engaged in during pre-pandemic times. Also, participants were asked “How much time do you currently spend engaging in high to moderate physical activity on average each day?” (*M*_during-pandemic_ = 38.30 min/day, *Median*_during-pandemic_ = 30.00, *SD* = 46.71), referring to their activity engagement at the time of the survey during pandemic restrictions of Spring/Summer 2020. Each evening, participants were asked how many steps they took that day (*M* = 5598.00 steps, *SD* = 5524.77). Participants were prompted to use step data from a personal activity watch or smartphone if they had access to one (a fitness device was used 42% of the time). Participants also reported minutes of moderate-to-vigorous physical activity during each evening questionnaire (*M* = 37.61 min, *SD* = 45.32).

Baseline typical (pre-pandemic times) and current (during pandemic times) moderate-to-vigorous physical activity data were examined and four participants were removed from these analyses as they reported values three standard deviations above the mean. Daily evening physical activity data were examined for outliers, and plots revealed no outliers in addition to the ranges for moderate-to-vigorous physical activity (range: 0–300 min) and number of steps (0–38,693 steps) being within reasonable limits; as such, no data points were removed. Instances where individuals reported 0 daily steps were considered as missing data.

#### Loneliness

Daily loneliness was assessed during each evening questionnaire for the 10-day study period (*M* = 24.72, *SD* = 27.72). Participants were asked to rate “How lonely did you feel today?.” Responses were recorded on a visual analog scale from 0 (Not at all) to 100 (Very Much).

### Control variables

Overall self-reported health, weekday, sex, and age were included as control variables in all analyses. Overall self-reported health was measured during the initial baseline questionnaire, where participants were asked to rate their health on a five-point scale (1 = Poor, 2 = Fair, 3 = Good, 4 = Very Good, 5 = Excellent; *M* = 3.32, *SD* = 0.94). Weekday was included as a dummy indexed variable to account for weekday versus weekend behavioral differences in physical activity. Sex was included as a dichotomous female/male variable. Age was included as a control variable and as a moderator variable in exploratory analyses discussed below.

### Statistical plan

Given the nested nature of the data underlying this project, a multilevel modelling approach with two levels was used: day level and person level. Moderate-to-vigorous physical activity and number of steps were examined as separate outcome variables, with loneliness as the predictor variable. To account for within-and between-person variation, random intercepts for loneliness and random slopes for daily loneliness were included. Age, overall loneliness, self-reported health, and day in study were grand mean centered, and sex and weekday were included as dichotomous variables. Please refer to Supplementary material, section 1 for model specifications.

## Results

Descriptive statistics and bivariate correlations are presented in Table 1. Bivariate analyses indicated that participants who self-identified as female reported less loneliness (*r* = −0.10, *p* < 0.01) and worse overall health (*r* = −0.09, *p* < 0.01) than those self-identifying as male in this sample. At the bivariate level, loneliness was significantly negatively associated with number of steps (*r* = −0.15, *p* < 0.001); the association between evening moderate-to-vigorous physical activity and loneliness did not reach statistical significance (*r* = −0.05, *p* = 0.18). Age was significantly positively associated with moderate-to-vigorous physical activity at the bivariate level (*r* = 0.12, *p* < 0.001). Using a fitness watch or smartphone to report daily number of steps was associated with reporting more daily steps (*r* = 0.29, *p* < 0.001) and more minutes of moderate-to-vigorous physical activity (*r* = 0.13, *p* < 0.001). Results from the t-test indicate that participants who used a fitness watch reported significantly more steps (*M* = 7,096, *SD* = 5,985) as compared to participants not using a fitness watch (*M* = 3,908, *SD* = 4,381), *t*(729) = −8.12, *p* < 0.001.

**Table 1:** Means, standard deviations, and intercorrelations of central study variables (*N* = 139).

Variable	Mean (*SD*)	1	2	3	4	5	6	7
1. Age	40.65 (18.37)							
2. Sex	0.80 (0.40)	−0.07*						
3. Overall health	3.32 (0.94)	0.03	−0.09**					
4. Weekday	–	0.00	0.00	0.05				
5. Evening loneliness	25.10 (27.88)	−0.06	−0.10**	−0.14***	−0.01			
6. Daily MVPA (mins.)	37.61 (45.32)	0.12***	−0.05	0.05	0.02	−0.05		
7. Daily steps	5,598 (5524.77)	0.01	0.03	0.09*	0.03	−0.15***	0.57***	
8. Use of fitness watch or smartphone	Yes: 42% No: 49% missing: 9%	−0.06	0.06	0.11*	−0.02	−0.13***	0.13***	0.29***

SD, standard deviation; MVPA, moderate-to-vigorous physical activity, measured in minutes. Sex was coded as 0 = male, 1 = female; Weekday was coded as 0 = weekday, 1 = weekend. Overall health was measured on a 1–5 scale (1 = poor, 2 = fair, 3 = good, 4 = very good, 5 = excellent). Evening loneliness was measured on a scale from 0 (not at all) – 100 (very much). Participants self-reported whether they used a smartphone or fitness watch to measure their number of steps and use of fitness watch or smartphone was coded as 0 = no fitness watch or smartphone, 1 = used a fitness watch or smartphone. *p < 0.05, **p < 0.01, ***p < 0.001.

Intraclass correlation coefficients (ICC’s) were calculated for the key study variables. In total, 57% of the variance in number of steps was due to between-person differences (ICC = 0.57) and 43% due to within-person differences. For moderate-to-vigorous physical activity, 38% of variance was due to between-person differences (ICC = 0.38) and 62% due to within-person differences. Between-person differences accounted for 62% of variance in loneliness (ICC = 0.62) and within-person differences accounted for 38% of variance in loneliness.

To better understand our data in the pandemic context, we examined self-reported differences in physical activity pre-pandemic versus during the pandemic at baseline. Consistent with other research emerging during the pandemic, there was a significant difference in moderate-to-vigorous physical activity engagement pre-pandemic (*M* = 54.76 min/day, *SD* = 52.37) and during the pandemic (*M* = 38.30 min/day, *SD* = 46.71); *t*(265) = −2.73, *p* = 0.007. Participants indicated that they engaged in 30% less moderate-to-vigorous physical activity during the pandemic.

### Loneliness and physical activity

**Figure 1:**
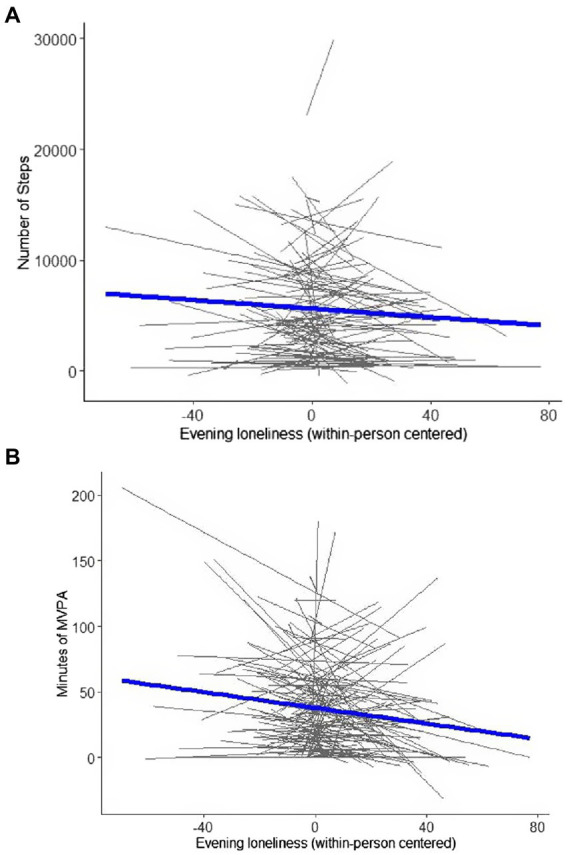
Model-implied within-person associations of daily evening loneliness with physical activity indicators (steps and MVPA). MVPA = moderate-to-vigorous physical activity. Number of steps **(A)** and moderate-to-vigorous physical activity **(B)** was higher when individuals felt less lonely than usual.

Results from separate analyses exploring how loneliness was associated with physical activity indices are displayed in Table 2, and results are illustrated in Figure 1. In line with our hypotheses, individuals who reported feeling lonelier on a particular day engaged in significantly less moderate-to-vigorous physical activity (*b* = −0.24, *p* = 0.007). There was a significant negative association between loneliness and daily number of steps (*b* = −18.42, *p* = 0.041). At the between-person level, there was no significant association between overall loneliness on moderate-to-vigorous physical activity engagement or number of steps. These results remained consistent after controlling for the use of a fitness watch or device in models with steps as the outcome variable (Supplementary Table S1).

**Table 2:** Results from multilevel models examining loneliness and physical activity (*N* = 139).

Predictors	Model 1 (Outcome: Steps)			Model 2 (Outcome: MVPA)		
	*B (SE)*	*CI*	*p*	*B (SE)*	*CI*	*p*
(Intercept)	5862.03 (1014.47)	3870.33–7853.73	<0.001***	38.14 (6.79)	24.81–51.47	<0.001***
Age	7.09 (21.25)	−34.63 – 48.82	0.739	0.30 (0.14)	0.02–0.57	0.034*
Sex	193.58 (959.79)	−1690.77 – 2077.94	0.840	−4.47 (6.38)	−17.08 – 8.08	0.483
Overall health	435.01 (438.40)	−425.70 – 1295.72	0.321	3.35 (2.81)	−2.16 – 8.87	0.233
Weekday	133.87 (295.73)	−446.73 – 714.48	0.651	1.34 (2.60)	−3.76 – 6.44	0.606
Daily loneliness	−18.42 (8.98)	−36.06 – −0.78	0.041*	−0.24 (0.09)	−0.42 – −0.07	0.007**
Average loneliness	−28.29 (17.27)	−62.19 – 5.61	0.102	0.07 (0.01)	−0.16 – 0.29	0.564
**Random effects**	***Variance***	***SD***	***Corr***	***Variance***	***SD***	***Corr***
Random intercept	1.69e+07	4109.43	–	747.16	27.33	–
Random slope	1.21e+07	3475.71	–	1217.59	34.89	
Intercept-slope correlation	928.70	30.47	−0.78	0.20	0.44	−0.63

B, unstandardized regression coefficient; CI, confidence interval; SE, standard error; MVPA, moderate-to-vigorous physical activity. Sex was coded as 0 = male, 1 = female. Weekday was coded as 0 = weekday, 1 = weekend. Age and overall health were centered to their means. Unstandardized estimates are reported for intercept and slope variance.

### Additional analyses

To further explore the associations between loneliness and physical activity, additional analyses examined the time-ordering of associations. We first explored morning loneliness as predicting same-evening reported physical activity controlling for between-person differences in age, health, weekday, and sex; however, no significant relationships were found. Time-lagged analyses were also run such that the relationship between previous day loneliness and next-day physical activity was examined with physical activity indicators as the main outcome variables. There were no significant results from the lagged analyses between loneliness and number of steps or moderate-to-vigorous physical activity at the within-or between-person levels. Age was explored as a moderating variable between loneliness and physical activity, but no significant cross-level interactions were found. In other words, the association between study variables did not significantly differ by age in the present sample. Finally, we conducted analyses controlling for household size to account for social isolation and did not find any significant differences in the reported main results.

All results and model specifications from these additional analyses are reported in Supplementary Tables S2–S6. In sum, this supports the robustness of our results indicating that evening-reported loneliness may be meaningfully associated with same-day physical activity across age groups, over and above social isolation.

## Discussion

### Physical activity as a health behavior

Despite evidence suggesting that physical activity may be an important coping strategy that mitigates the impact of mandated social distancing on wellbeing (Manuel et al., 2021), research indicates that physical activity engagement has decreased during the pandemic (Adams et al., 2021; Maltagliati et al., 2021). Results from the current study indicating that adults reported a significant decrease in moderate-to-vigorous physical activity compared to pre-pandemic times highlights the importance of supporting physical activity engagement for individuals now, more than ever. Pandemic circumstances may have incurred more loneliness for individuals who do not typically report feeling lonely (O’Sullivan et al., 2021); however, loneliness is an experience that is relevant to almost all individuals at one point or another even during “normal” life (Hawkley and Cacioppo, 2010; Surkalim et al., 2022). The repeated daily life assessment design of the current study allowed us to examine the associations between barriers to physical activity and physical activity at the within-person level. With significant amounts of day-to-day variation in physical activity behaviors within our sample, we were able to gain a snapshot into our participants daily lives that may be targeted in interventions that are feasible at the day-level.

Contrary to pre-pandemic research, we found that age was significantly positively associated with moderate-to-vigorous physical activity in the present sample at the bivariate level. Although this finding is not consistent with previous work, it does raise an interesting question about how physical activity behaviors may have changed for different age groups during the pandemic. One potential reason for why older adults may have engaged in more physical activity than younger adults may be that their everyday physical activity routines may have been less impacted by social distancing measures than those of younger and middle-aged adults. For example, adults between the ages of 18 and 54 years old make up 60.60% of gym members around the world (The Global Health and Fitness Association, 2022) and older adults report walking, gardening, and home exercise as the top physical activities they engage in (The Canadian Fitness and Lifestyle Research Institute, 1996). As a result, older adults may have been less impacted by the closing of fitness facilities and found their routines less disrupted. Also, older adults are often retired and may not have experienced a large increase in sedentary behavior due to classes being moved to a virtual environment and middle-aged adults with children found themselves balancing work, homeschooling and child care, which may have left little room to carve out intentional physical activity in their day-to-day lives.

### Loneliness and physical activity

Our results indicated a significant negative within-person relationship between evening-reported loneliness and concurrent evening-reported physical activity behaviors from that same day; no significant between-person relationships between these main study variables were found. One possible explanation for not finding significant between-person results may be the similar restrictions Canadians experienced during the pandemic. As the pandemic unfolded, fitness facilities and in-person exercise opportunities slowed, and sometimes, entirely stopped. Because of this, individuals may have found themselves in a similar situation to their peers, which may have restricted between-person variance in physical activity behaviors. Interestingly, our results indicate that, even in these unique circumstances, the time-varying relationship between loneliness and physical activity still matters. From an intervention perspective, this may support targeting loneliness on a day-to-day basis since intensive loneliness interventions are often time and resource intensive (Osborn et al., 2021). For example, it may be more feasible to target loneliness on a day-to-day basis by encouraging individuals to engage in activities that make them feel less lonely, such as a phone call with a friend, rather than providing broad statements on how to reduce loneliness such as establishing new close friend connections (Osborn et al., 2021). Experimental research has also found that interventions were most successful when they involved a cognitive behavioral therapy or psychological reframing component (Masi et al., 2011) and recent research has found merit in internet use to support communication with friends and reduce loneliness (Sharabi and Margalit, 2011). These interventions may be explored as ways to target daily loneliness and the subsequent health behaviors that are associated with loneliness. This may be especially relevant during times of mandated social-distancing, but also at other times when individuals experience greater feelings of loneliness throughout their life such as during a health crisis or during turbulent life or world events.

### Alternative explanations

In our additional analyses exploring the time-ordering of our results, we found that after accounting for between-person differences in age, health, weekday and sex, daily reported morning loneliness did not predict daily physical activity, despite significant effects between evening reported loneliness and physical activity behaviors. This may suggest that momentary appraisals of loneliness are not sufficient for predicting physical activity outcomes, and that reflective self-reported assessment of how lonely one felt on a particular day may be more powerful in predicting physical activity behaviors.

We explored the robustness of our results by examining time-lagged associations between loneliness and physical activity indices. There were no significant within-person results from these lagged analyses, suggesting that loneliness from the previous day does not significantly predict next-day physical activity behaviors. This may be explained with the understanding that affective states, such as loneliness, have a state component which may fluctuate by day (Hawkley et al., 2009; van Roekel et al., 2018) such that each day’s loneliness appraisals serve as a better predictor of same-day physical activities. This may suggest that loneliness be targeted on a finer time scale, such that interventions target daily loneliness as opposed to overall loneliness, which is the focus of most current interventions (Osborn et al., 2021). In the larger loneliness literature, interventions that address negative cognitions around loneliness by promoting activities like personal contact, counselling and community engagement show the strongest evidence for effective intervention (Osborn et al., 2021). Given the risk of social isolation creating a “loneliness loop” whereby individuals continue to distance themselves from others and feed their negative cognitions around loneliness (Hawkley and Cacioppo, 2010), momentary intervention that interrupts this loop may be an alternative approach to explore. As discussed above, small-scale daily behaviors to interrupt loneliness (such as calling a friend) may have the potential to serve as feasible interventions to address the significant time-varying associations between loneliness and physical activity.

Further, age was explored as a moderating variable between loneliness and physical activity. We did not find any significant cross-level interactions between age and our main predictor variables. In other words, the association between study variables did not significantly differ by age. It is important to keep in mind that our sample was relatively homogeneous, and only included 29 participants aged 60 years or older and 10 participants under the age of 20.

### Limitations and future directions

It is important to note that the current study was conducted entirely online given the COVID-19 restrictions. Because of these circumstances, participants who expressed interest in the study may be considered a convenience sample, and our sample was relatively homogeneous, with the majority of respondents self-identifying as female, white, highly educated, and relatively healthy. The current study uses a correlational design and cannot address the direction of the relationship between physical activity and loneliness or the underlying mechanism of this relationship. For example, the loneliness-physical activity association may be bi-directional such that physical activity promotes social engagement and thus decreases same-day feelings of loneliness.

Physical activity was assessed via self-report, without the use of “objective” measurement instruments such as accelerometers, which provide data that can later be examined in-lab. As discussed above, there was a positive relationship between the use of a fitness watch or device in reporting number of steps and physical activity reported in the evening. This may be indicative of individuals who use these devices having elevated health conscientiousness and thus engaging in more physical activity. Alternatively, it may point to the fact that individuals may not be accurate in reporting their physical activity engagement. Literature suggests that individuals often under-report daily activities such as gardening or running errands as physical activity, but may also significantly over-report moderate and vigorous physical activity (Prince et al., 2008).

In future research, we would ideally include the use of an activity tracker to gain better insight into differences in daily “objective” vs. “subjective” number of steps and moderate-to-vigorous physical activity measures. In addition, we recognize the importance of understanding how key demographic characteristics, such as parenting, employment, or relationship status may influence physical activity behaviors. For example, parents balancing childcare, working from home, and home-schooling during the pandemic would have faced unique barriers to engaging in regular physical activity. We aim to incorporate a more comprehensive understanding of these sample characteristics in future study designs.

### Conclusion

The present study provides evidence of loneliness as a meaningful barrier to physical activity for adults during the COVID-19 pandemic. This main finding builds on past literature indicating that loneliness and physical activity have a negative association at the between-person level; and it extends these results to a community sample using repeated daily life assessments. By exploring potential barriers to physical activity in the daily lives of adults, we aimed to shed light on potential targets of intervention that may be particularly useful during times of challenge. Findings from this research may extend to other particularly challenging times, where “normal” social contact is not possible. This builds on literature highlighting the importance of social connectedness and social interaction for health and wellbeing across the lifespan.

## Data availability statement

The raw data supporting the conclusions of this article will be made available by the authors, without undue reservation.

## Ethics statement

The studies involving human participants were reviewed and approved by the Behavioral Research Ethics Board of the University of British Columbia. The patients/participants provided their written informed consent to participate in this study.

## Author contributions

CH designed and directed the project and supervised the work. DG and TP helped design the project and provided feedback. TB created a theoretical framework, preregistered hypotheses, performed data analysis, and wrote the manuscript. YC contributed to the data analysis and together with EZ provided feedback. All authors commented on the manuscript and contributed to the final version of the manuscript.

## Funding

We gratefully acknowledge support from the Canada Graduate Scholarship – Master’s program (CGS-M) to TB and the Canada Research Chairs (CRC) Program to CH. TP acknowledges support from the University of Zurich (Forschungskredit, grant # FK-21-078), EZ acknowledges support from UBC’s Four Year Doctoral Fellowship, and YC acknowledges support from UBC’s Four Year Doctoral Fellowship.

## Conflict of interest

The authors declare that the research was conducted in the absence of any commercial or financial relationships that could be construed as a potential conflict of interest.

## Publisher’s note

All claims expressed in this article are solely those of the authors and do not necessarily represent those of their affiliated organizations, or those of the publisher, the editors and the reviewers. Any product that may be evaluated in this article, or claim that may be made by its manufacturer, is not guaranteed or endorsed by the publisher.
