# Supplementary material for: Time-varying associations between loneliness and physical activity: Evidence from repeated daily life assessments in an adult lifespan sample
Source: Front Psychol. 2023 Jan 26;13:1021863. doi: 10.3389/fpsyg.2022.1021863 (PMC9909092; doi:10.3389/fpsyg.2022.1021863)

Supplementary Material

1 Model Specification

1.1 Variables names

ParticipantID = Participant ID number

Birthdate= Birthdate

age= Age

sex= gender

weekend= Whether questionnaire was completed on weekday (0) or weekend (1)

ethn_bs= self-reported ethnicity

piyh_bs= number of people in household

edu_bs= Education level

heth_bs= Self-reported health at baseline

mstat_bs= marital status

employ= employment status

noc_bs= number of children you are raising

mvpa_typical= Typical MVPA engagement (pre-pandemic)

mvpa_current= MVPA engagement during the pandemic

isolated_bs= Self-reported isolation at baseline (How isolated have you felt in the past month?)

lonely_bs= Self-reported loneliness at baseline (How lonely have you felt in the past month?)

lonely_eve= Daily evening loneliness (How lonely did you feel today?; 0-100 scale)

lonely_morn= Daily morning loneliness (How lonely do you feel?; 0-100 scale)

mvpa_eve= Daily evening MVPA

stps_eve= Daily evening number of steps

fw_eve= Use of a fitness watch to track steps (Did you use a fitness watch today?; 0=no, 1=yes)

1.2 Code for centering

```
# Grand mean centering: Age, health status, day in study, household size (variable.c = grand-mean centered)
df <- df %>% mutate(age.c = df$age - mean(df$age, na.rm=T))
df <- df %>% mutate(hlth.c = df$heth_bs - mean(df$heth_bs, na.rm=T))
df <- df %>% mutate(house.c = df$piyh_bs - mean(df$piyh_bs, na.rm=T))

# Creating Person Means: Pain, Loneliness, Steps, MVPA (variable.pmean = person-mean)
df = df %>% group_by(ParticipantID) %>% dplyr:: mutate(pain_eve.pmean = mean(pain_eve, na.rm = TRUE))
df = df %>% group_by(ParticipantID) %>% dplyr::mutate(lonely_eve.pmean = mean(lonely_eve, na.rm = TRUE))
df = df %>% group_by(ParticipantID) %>% dplyr::mutate(stps_eve.pmean = mean(stps_eve, na.rm = TRUE))
df = df %>% group_by(ParticipantID) %>% dplyr::mutate(mvpa_eve.pmean = mean(mvpa_eve, na.rm = TRUE))

# Person Mean Centering (variable.pmc = person-mean centered)
df <- df %>% group_by(ParticipantID) %>% dplyr::mutate(pain_eve.pmc = pain_eve - mean(pain_eve, na.rm=T))
df <- df %>% group_by(ParticipantID) %>% dplyr::mutate(lonely_eve.pmc = lonely_eve - mean(lonely_eve, na.rm=T))
df <- df %>% group_by(ParticipantID) %>% dplyr::mutate(stps_eve.pmc = stps_eve - mean(stps_eve, na.rm=T))
df <- df %>% group_by(ParticipantID) %>% dplyr::mutate(mvpa_eve.pmc = mvpa_eve - mean(mvpa_eve, na.rm=T))
```

1.3 Model 1: Evening-reported loneliness predicting same-day steps and MVPA

```
# Outcome variable: number of steps or MVPA
# Predictor variable: daily evening loneliness
# Controls: age.c, hlth.c, weekend, sex
# Person-means: lonely_eve.pmean
# Person-mean centering: lonely_eve.pmc

model_1A <- lmer(stps_eve  ~ 1 + age.c + hlth.c + weekday + sex + lonely_eve.pmean +
        lonely_eve.pmc + (1 + lonely_eve.pmc|ParticipantID), data = df, REML=TRUE,
        control=lmerControl(optimizer="bobyqa", optCtrl=list(maxfun=2e5)))

model_1B <- lmer(mvpa_eve  ~ 1 + age.c + hlth.c + weekday + sex + lonely_eve.pmean +
        lonely_eve.pmc + (1 + lonely_eve.pmc|ParticipantID), data = df, REML = TRUE,
        control=lmerControl(optimizer="bobyqa", optCtrl=list(maxfun=2e5)))
```

1.4 Model 2: Morning loneliness predicting evening steps and MVPA

```
Model_2A <- lmer(stps_eve  ~ 1 + age.c + hlth.c + weekday + sex +lonely_morn.pmean +
    lonely_morn.pmc +  (1 + lonely_morn.pmc|ParticipantID), data =
    bs_morn_eve_removed, REML = TRUE, control=lmerControl(optimizer="bobyqa",
    optCtrl=list(maxfun=2e5)))

Model_2B <- lmer(mvpa_eve  ~ 1 + age.c + hlth.c + weekday + sex + lonely_morn.pmean +
    lonely_morn.pmc + (1 + lonely_morn.pmc|ParticipantID), data =
    bs_morn_eve_removed, REML = TRUE, control=lmerControl(optimizer="bobyqa",
    optCtrl=list(maxfun=2e5)))
```

1.5 Model 3: Lagged effects – Previous day evening loneliness predicting next day PA

```
Model_3A <- lmer(lvar_stps  ~ 1 + age.c + hlth.c + weekday + sex + lonely_eve.pmean +
    lonely_eve.pmc + (1 + lonely_eve.pmc|ParticipantID), data = df_lagged_analyses,
    REML = TRUE, control=lmerControl(optimizer="bobyqa", optCtrl=list(maxfun=2e5)))

Model_3B <- lmer(lvar_mvpa  ~ 1 + age.c + hlth.c + weekday + sex + lonely_eve.pmean +
    lonely_eve.pmc + (1 + lonely_eve.pmc|ParticipantID), data = df_lagged_analyses,
    REML = TRUE, control=lmerControl(optimizer="bobyqa", optCtrl=list(maxfun=2e5)))
```

2 Supplementary Tables

2.1 Table S1

Results from Multilevel Models Examining Loneliness and Number of Steps, Controlling for Use of Fitness Device (N = 139)

Predictors	*B (SE)*	*CI*	*p*
(Intercept)	9976.30 (1227.04)	3324.00 – 4511.21	< .001***
Age	20.39 (20.41)	-23.08 – 56.41	.320
Sex	-255.03 (917.97)	-2015.01 – 1693.35	.782
Overall Health	375.83 (417.82)	-457.08 – 1192.20	.370
Weekday	112.20 (286.67)	-497.32 – 675.26	.696
Use of fitness device	2792.07 (491.56)	-3797.90 – -1791.53	< .001***
Daily Loneliness	-20.00 (8.50)	-37.70 – 12.96	.023*
Average Loneliness	-17.94 (16.45)	-52.40 – -1.83	.278
Daily Loneliness x Use of fitness device	16.02 (23.73)	-35.30 – 63.41	.500
Random Effects	*Variance*	*SD*	*Corr*
Random Intercept	1.51e+07	3884.06	-
Random Slope	1.16e+07	3406.95	-
Intercept-slope correlation	740.10	27.21	-0.67

Note. B = unstandardized regression coefficient; *CI* = confidence interval, *SE* = standard error, MVPA = moderate-to-vigorous physical activity. Sex was coded as 0 = male, 1 = female. Weekday was coded as 0 = weekday, 1 = weekend. Use of fitness device was coded as: 0 = did not use a fitness device to record number of steps, 1 = used a fitness device to record number of steps. Age and overall health were centered to their means. Unstandardized estimates are reported for intercept and slope variance.

2.2 Table S2

Results of Multilevel Models with Daily Morning Loneliness Predicting Same-Day Physical Activity

	**Model 1 (Outcome: Next-Day Loneliness)**		**Model 2 (Outcome: Next-Day Loneliness)**	
Predictors	*B (SE)*	*p*	*B (SE)*	*p*
(Intercept)	6575.14 (1059.56)	< .001***	40.13 (7.02)	< .001***
Age	-8.32 (22.52)	.713	0.33 (0.15)	.031*
Sex	-774.37 (1028.05)	.453	–6.58 (6.85)	.339
Overall Health	167.50 (470.55)	.723	-0.49 (3.03)	.871
Daily Evening Steps	3.90 (8.65)	.653	-	-
Average Evening Steps	-33.94 (17.58)	.056	-	-
Daily Evening MVPA	-	-	-0.03 (0.08)	.653
Average Evening MVPA	-	-	0.00 (0.12)	.975

Random Effects	*Variance*	*SD*	*Corr*	*Variance*	*SD*	*Corr*
Random Intercept	1.68e+07	4102.69	-	689.20	26.25	-
Random Slope	1.12e+07	3351.34	-	1125.00	33.53	-
Intercept-slope correlation	422.50	20.56	1.00	0.02	0.12	1.00

Note. B = unstandardized regression coefficient; *CI* = confidence interval, *SE* = standard error, MVPA = moderate-to-vigorous physical activity. Sex was coded as 0 = male, 1 = female. Age and overall health were centered to their means. Unstandardized estimates are reported for intercept and slope variance.

2.3 Table S3

Time-Lagged Results of Multilevel Models with Previous Day Loneliness Predicting Next-Day Physical Activity

	**Model 1 (Outcome: Steps)**			**Model 2 (Outcome: MVPA)**		
Predictors	*B (SE)*	*CI*	*p*	*B (SE)*	*CI*	*p*
(Intercept)	6252.75 (1118.41)	3519.27 – 4842.30	< .001***	36.56 (7.26)	21.81 – 50.83	< .001***
Age	-7.83 (23.42)	-54.92 – 38.38	.739	0.34 (0.15)	0.05 – 0.65	.026*
Sex	-525.98 (1073.31)	-2731.69 – 1410.21	.625	–3.98 (6.94)	-16.67 – 10.48	.568
Overall Health	86.68 (499.62)	-868.36 – 990.82	.863	-0.47 (3.12)	-6.93 – 5.57	.881
Weekday	460.68 (343.84)	-185.84 – 1188.37	.181	2.82 (3.08)	-3.71 – 8.79	.360
Daily Loneliness	2.01 (9.49)	-17.50 – 18.62	.832	0.04 (0.09)	-0.14 – 0.21	.686
Average Loneliness	-30.47 (18.62)	-70.18 – 5.55	.105	0.02 (0.12)	-0.21 – 0.26	.855
Random Effects	*Variance*	*SD*	*Corr*	*Variance*	*SD*	*Corr*
Random Intercept	1.71e+07	4139.25	-	658.20	25.66	-
Random Slope	1.15e+07	3387.06	-	1165.00	34.13	-
Intercept-slope correlation	77.74	8.82	1.00	0.02	0.13	1.00

Note. B = unstandardized regression coefficient; *CI* = confidence interval, *SE* = standard error, MVPA = moderate-to-vigorous physical activity. Sex was coded as 0 = male, 1 = female. Weekday was coded as 0 = weekday, 1 = weekend. Age and overall health were centered to their means. Unstandardized estimates are reported for intercept and slope variance.

2.4 Table S4

Results from Multilevel Models With Previous Day Physical Activity Predicting Next-Day Loneliness

Predictors	*B (SE)*	*CI*	*p*
(Intercept)	31.12 (5.82)	3324.00 – 4511.21	< .001***
Age	-0.21 (0.12)	-23.08 – 56.41	.090
Sex	-6.42 (5.48)	-2015.01 – 1693.35	.244
Overall Health	-6.62 (2.52)	-457.08 – 1192.20	.010*
Weekday	4.71 (1.89)	-497.32 – 675.26	.013*
Daily steps	1.15e-04 (3.11e-04)	-3797.90 – -1791.53	.713
Average steps	-1.42e-03 (6.59e-04)	-37.70 – 12.96	.340
Daily MVPA	-0.08 (0.52)	-52.40 – -1.83	.883
Average MVPA	0.16 (0.10)	-35.30 – 63.41	.110
Random Effects	*Variance*	*SD*	*Corr*
Random Intercept	419.70	20.49	-
Random Slope	270.70	16.45	-
Intercept-slope correlation (steps)	3.50e-09	5.91e-05	-1.00
Intercept-slope correlation (MVPA)	24.00	4.90	0.21

Note. B = unstandardized regression coefficient; *CI* = confidence interval, *SE* = standard error, MVPA = moderate-to-vigorous physical activity. Sex was coded as 0 = male, 1 = female. Weekday was coded as 0 = weekday, 1 = weekend. Age and overall health were centered to their means. Unstandardized estimates are reported for intercept and slope variance.

2.5 Table S5

Results from Multilevel Models Examining Age as a Moderator for the Relationship Between Loneliness and Physical Activity

	Coefficient	Standard Error	*p* value
Cross-Level Interactions Predicting Daily Steps			
Age x Loneliness	0.77	0.51	.137
Cross-level Interactions Predicting Daily MVPA			
Age x Loneliness	0.005	0.005	.297

2.6 Table S6

Results from Multilevel Models with Loneliness Predicting Physical Activity, Controlling for Household Size, Relationship Status, and Raising Children

	**Model 1 (Outcome: Steps)**			**Model 2 (Outcome: MVPA)**		
Predictors	*B (SE)*	*CI*	*p*	*B (SE)*	*CI*	*p*
(Intercept)	5902.67 (1200.76)	3359.81 – 8169.58	< .001***	35.63 (7.83)	19.65 – 50.76	< .001***
Age	-7.74 (29.55)	-60.69 – 51.03	.794	0.38 (0.19)	0.03 – 0.75	.046*
Sex	117.81 (975.84)	-1633.39 – 2156.74	.904	–3.44 (6.53)	-17.30 – 9.39	.599
Overall Health	511.59 (440.61)	-260.57 – 1400.82	.248	3.67 (2.83)	-2.12 – 9.08	.197
Weekday	121.49 (289.49)	-455.67 – 673.96	.675	1.37 (2.60)	-3.97 – 5.52	.599
Number of People in Household	32.12 (283.51)	-588.65 – 606.53	.910	2.26 (1.80)	-1.12 – 5.53	.211
Relationship Status	-684.08 (1102.61)	-2779.59 – 1264.50	.536	2.05 (6.93)	-11.33 – 16.06	.767
Raising Children	824.27 (1331.67)	-2070.04 – 3249.56	.537	5.29 (8.56)	-13.15 – 23.61	.537
Daily Loneliness	-19.65 (8.56)	-36.05 – -2.27	.026*	-0.25 (0.09)	-0.44 – -0.60	.008**
Average Loneliness	-28.62 (17.12)	-62.88 – 5.92	.097	0.07 (0.12)	-0.20 – 0.30	.568
Random Effects	*Variance*	*SD*	*Corr*	*Variance*	*SD*	*Corr*
Random Intercept	1.73e+07	4155.00	-	747.93	27.35	-
Random Slope	1.19e+07	3452.00	-	1218.04	34.90	-
Intercept-slope correlation	784.00	28.00	-0.78	0.21	0.45	-0.62

Note. B = unstandardized regression coefficient; *CI* = confidence interval, *SE* = standard error, MVPA = moderate-to-vigorous physical activity. Sex was coded as 0 = male, 1 = female. Weekday was coded as 0 = weekday, 1 = weekend. Relationship was coded as 0 = not single, 1 = single. Raising children was coded as 0 = not raising children, 1 = raising children. Age and overall health were centered to their means. Unstandardized estimates are reported for intercept and slope variance.

3 Supplementary Figures

Figure 1

Distribution of Self-Reported Daily MVPA During Pre-Pandemic Times (N=135)

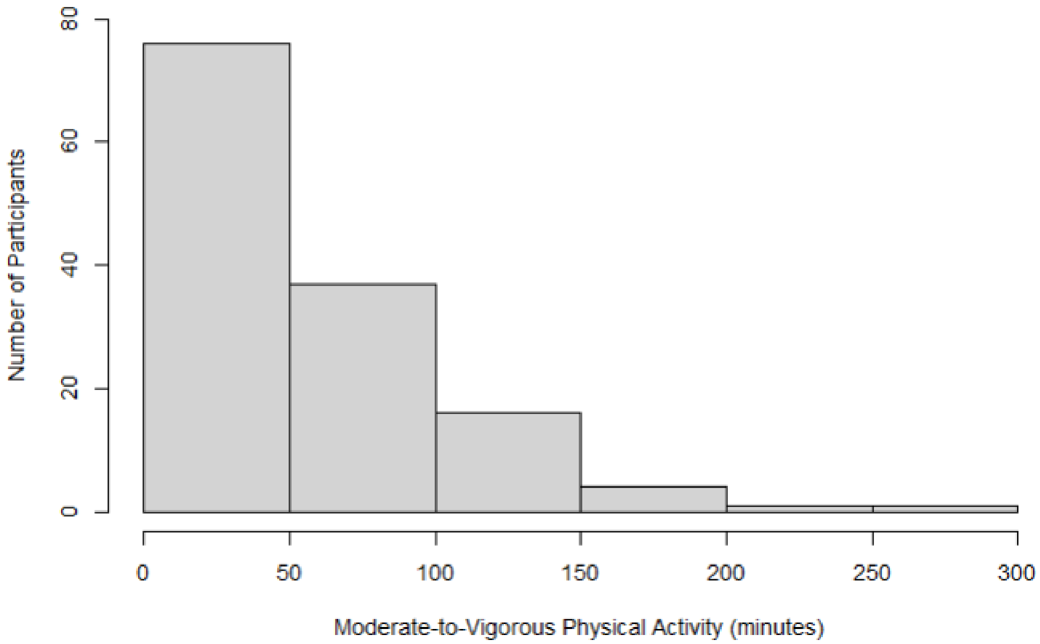

Figure 2

Distribution of Self-Reported Daily MVPA During Pandemic Times (N=135)

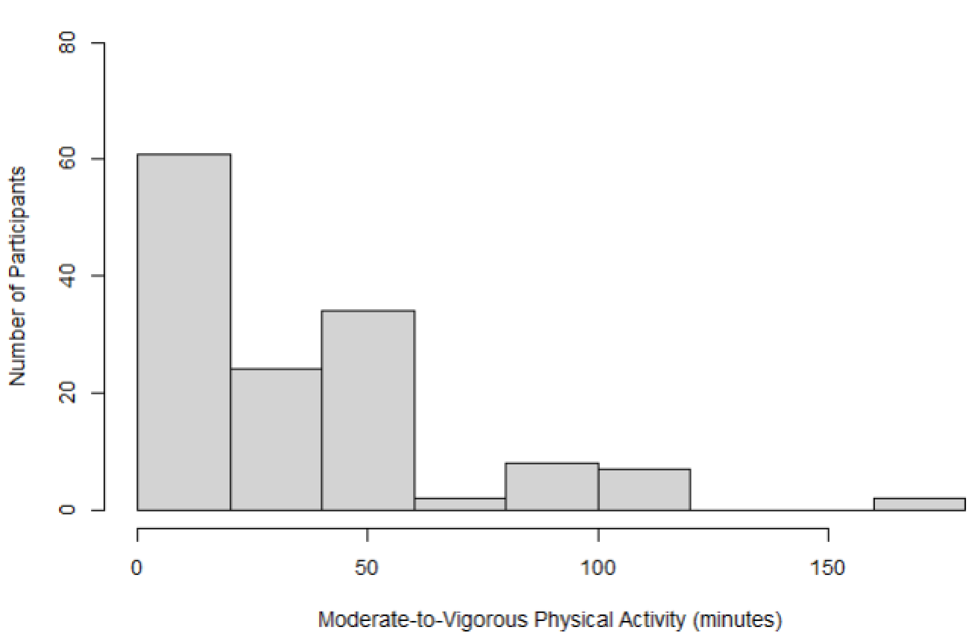

Supplement: Supplementary file 1 [file Data_Sheet_1.PDF]
